# The value of Ki67 for the diagnosis of LSIL and the problems of p16 in the diagnosis of HSIL

**DOI:** 10.1038/s41598-022-11584-z

**Published:** 2022-05-09

**Authors:** Jixuan Liu, Sanmei Su, Yafang Liu

**Affiliations:** Department of Pathology, The First Hospital of Jilin University, Changchun, 130021 Jilin China

**Keywords:** Cancer, Oncology

## Abstract

p16 and Ki67 are immunohistochemical markers related to cervical squamous intraepithelial lesions. p16 has been widely used to assist in the diagnosis of high-grade squamous intraepithelial lesions. However, a conclusion about the role of Ki67 in the diagnosis of squamous intraepithelial lesions has not been established. The aim of this study was to analyze the role of p16 and Ki67 immunohistochemical staining in assisting cervical squamous intraepithelial lesions. This study performed immunohistochemical staining for p16 and Ki67 on 1024 cervical biopsy specimens at our hospital to compare the differences between p16 and Ki67 in different cervical lesions using the chi-squared test and Fisher’s exact test. This study also evaluated the value of Ki67 for the diagnosis of low-grade squamous intraepithelial lesions (LSILs) using the receiver operating characteristic curve. The results indicated that Ki67 had high specificity and sensitivity in distinguishing LSIL from normal cervix. p16 was diffusely and strongly positive in some LSILs, and some problems were encountered in the interpretation of p16 staining. Therefore, we believe that Ki67 can be used as an immunohistochemical marker to help in the diagnosis of LSIL, to distinguish lesions that are difficult to morphologically determine and to avoid misdiagnosis. The practical application of p16 staining is still problematic. It may be necessary to find other auxiliary means to distinguish this small proportion of cervical lesions.

## Introduction

In 2012, the LAST project published a consensus and suggested that cervical squamous intraepithelial lesions caused by human papillomavirus (HPV) should be divided into two categories: low-grade cervical squamous intraepithelial lesions (LSIL) and high-grade cervical squamous intraepithelial lesions (HSIL). This article suggests that cervical squamous intraepithelial lesions should be diagnosed using histological morphology. When LSIL and HSIL are difficult to distinguish by morphology, immunohistochemistry can be used to assist in the diagnosis^1^. In distinguishing between LSIL and HSIL, p16 positivity was defined as a combination of strong positive diffuse nuclear and cytoplasmic staining in more than 2/3 of the layers of the cervical squamous epithelium^2^. However, in daily clinical practice, the distinction of HSIL and LSIL using p16 immunohistochemical staining is difficult in some special cases. A meta-analysis of 61 published articles on p16 immunohistochemical staining showed that p16 exhibited diffuse positive staining in 2% of normal cervical squamous epithelium, 38% of low-grade cervical intraepithelial neoplasia (CIN1), 62% of CIN2 and 82% of CIN3^3^. Ki67 is an a nuclear protein that is considered to be related to cell proliferation. Some studies suggest that Ki67 is closely related to the progression of cervical squamous intraepithelial lesions^4,5^. However, few studies have been published on the relationship between Ki67 expression and the degree of pathology in cervical squamous intraepithelial lesions. This study retrospectively analysed the histology of all cervical biopsy specimens at our hospital from January 2019 to January 2021 and performed p16INK4a and Ki67 immunohistochemistry in all biopsy specimens. The aim was to evaluate p16INK4a and Ki67 expression in normal cervix, LSIL and HSIL and to evaluate the role of p16INK4a and Ki67 in the diagnosis of LSIL and HSIL in biopsy specimens.

## Method

### Study design and patient selection

From January 2019 to January 2021, among the women who presented to the Obstetrics and Gynaecology Clinic of the First Hospital of Jilin University, all who underwent biopsy were retrospectively included in this study.

### Histological evaluation and immunohistochemical detection of p16 and Ki67

**Figure 1:**
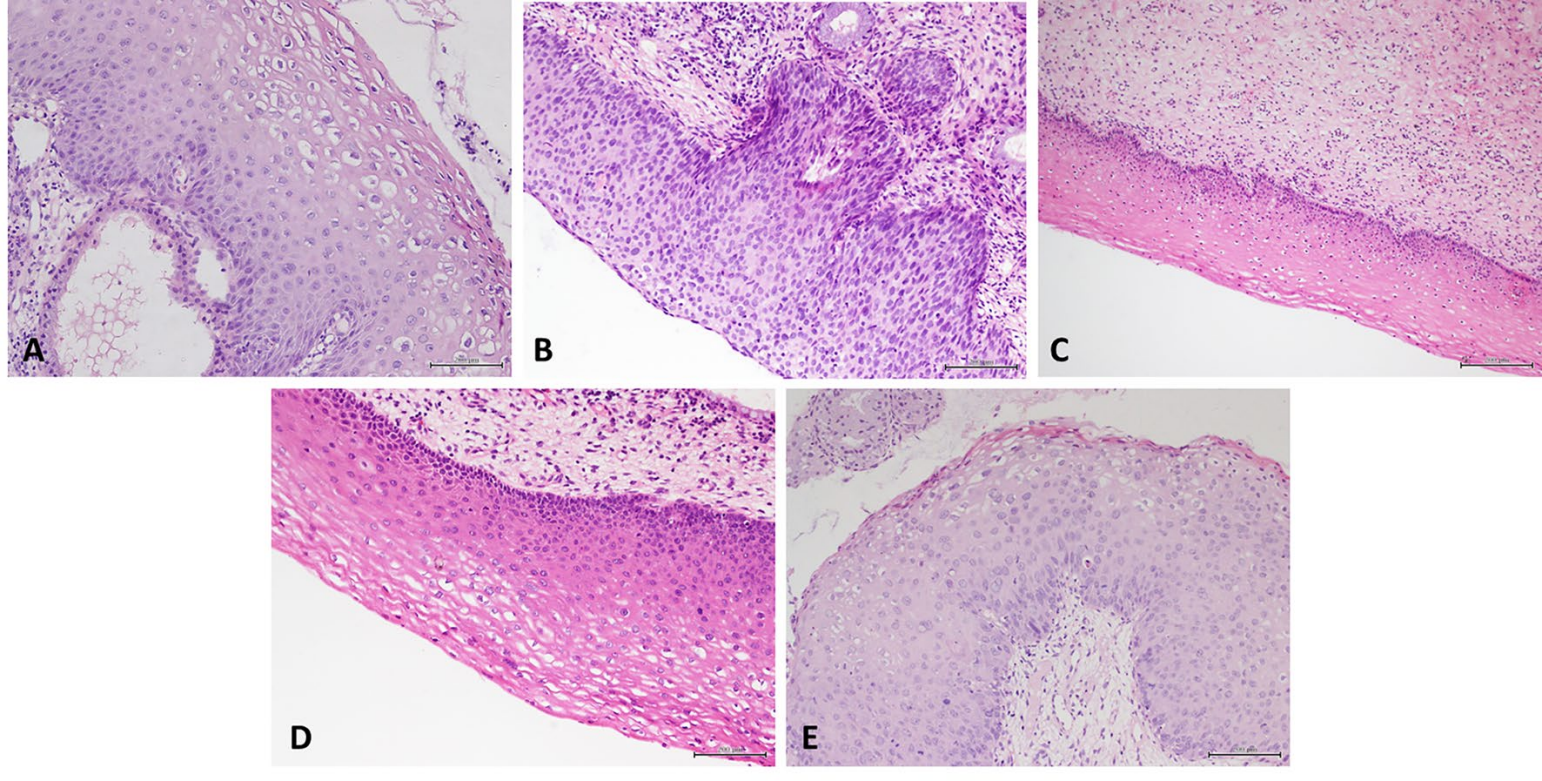
(**A**) Histological image of LSIL (**B**) Histological image of LSIL (**C**) Histological image of the cervix not infected by HPV (**D**) Cervical biopsy tissue showed some suspicious morphological features of LSIL (**E**) The morphology of cervical biopsy is between LSIL and HSIL.

All histological samples were fixed in 10% neutral-buffered formalin and embedded in paraffin according to routine procedures. Haematoxylin and eosin-stained slides of all biopsy samples were reviewed by two pathologists(Consultant) and classified according to the criteria outlined by the LAST project^1^. This study included 1024 patients who underwent cervical biopsy. According to the suggestion of the LAST project group, 1024 biopsy specimens were graded according to their histological morphology^1^. The morphological characteristics of LSIL (Fig. 1A) include the following: ①Abnormalities of squamous cell nuclei: such as nuclear enlargement, irregular nuclear membrane and high nuclear-to-cytoplasmic ratio; ②The cytoplasm of the upper 2/3 layer squamous cells gradually matured; ③ mitotic figures limited to the lower third of the epithelium; and or ④ koilocytosis. The morphological characteristics of HSIL (Fig. 1B) include the following: ①Abnormalities of squamous cell nuclei; ②Undifferentiation of cytoplasm in the upper 2/3 layer of squamous epithelium; ③mitotic figures can occur in the upper 2/3 layer of squamous epithelium; ④pathological mitosis at any level. When no morphological features of LSIL or HSIL were observed, the lesion was classified as normal cervix (Fig. 1C). However, some of the biopsy specimens exhibited features between LSIL and normal cervix and exhibited some morphological features suspected to be LSIL (Fig. 1D). However, a small portion of the specimens, whose morphology was between LSIL and HSIL, demonstrated uncertain cytoplasmic differentiation in the middle or upper third of the epithelium (Fig. 1E). Therefore, we divided these cervical biopsies into five groups: normal, uncertain LSIL, LSIL, HSIL and uncertain HSIL. According to the histological classification, all 1024 specimens were classified as normal (217 cases), uncertain LSIL (68 cases), LSIL (207 cases), uncertain HSIL (128 cases) and HSIL (404 cases) (Tables 1 and 2).

**Table 1:** Correlation of p16 immunostaining patterns with histological diagnosis.

p16 Patterns	Normal	Uncertain LSIL	LSIL	Uncertain HSIL	HSIL	Total
Negative	158	57	118	12	4	349
Weakly positive	59	9	79	44	0	191
Diffusely and strongly positive	0	2	10	72	400	484
Total	217	68	207	128	404	1024

**Table 2:** Correlation of Ki67 immunostaining patterns with histological diagnosis.

Ki67 Patterns	Normal	Uncertain LSIL	LSIL	Uncertain HSIL	HSIL	Total
Basal layer and parabasal layer	209	47	15	8	0	279
The upper 2/3 of the squamous epithelium	8	21	192	120	404	745
Total	217	68	207	128	404	1024

Immunohistochemistry was performed using an anti-p16 mouse monoclonal antibody (clone 16P04/JC2; GeneTech, Shanghai, China) and an anti-Ki67 mouse monoclonal antibody (clone UMAB107; ZSGB-BIO, Beijing, China). Immunohistochemistry was performed using the Autostainer Link 48 automated system (Dako Co., Carpinteria, CA, USA) and the EnVision system (Dako). p16 expression is divided into three types: focally positive, diffusely and strongly positive and negative. Two types of Ki67 expression have been defined, namely, basal and parabasal layer positivity and positivity in the upper two-thirds of the squamous epithelium.

### Data analysis

Fisher’s exact test and chi-squared test were used to assess the correlation between the histological diagnosis and the immunohistochemical expression patterns of p16 and Ki67. Histological diagnosis was employed as a standard reference. Therefore, we excluded those cases with uncertain histological diagnosis, including 68 cases of indeterminate LSIL and 128 cases of indeterminate HSIL. Statistical analyses were conducted using SPSS 22 (SPSS, Chicago, IL, USA). A two-sided *P*-value < 0.05 was considered significant.

### Ethics approval and consent to participate

The patient provided informed consent, and the article was approved by the Ethical Committee of the First Hospital of Jilin University in Changchun, China. We obey the principles of the 1983 Declaration of Helsinki. In other words, all of experiments in this paper obey this principle.

### Consent for publication

Written informed consent for the publication of the clinical details and images was obtained from the patient.

## Result

### The expression of p16 in various histological diagnosis situations

**Figure 2:**
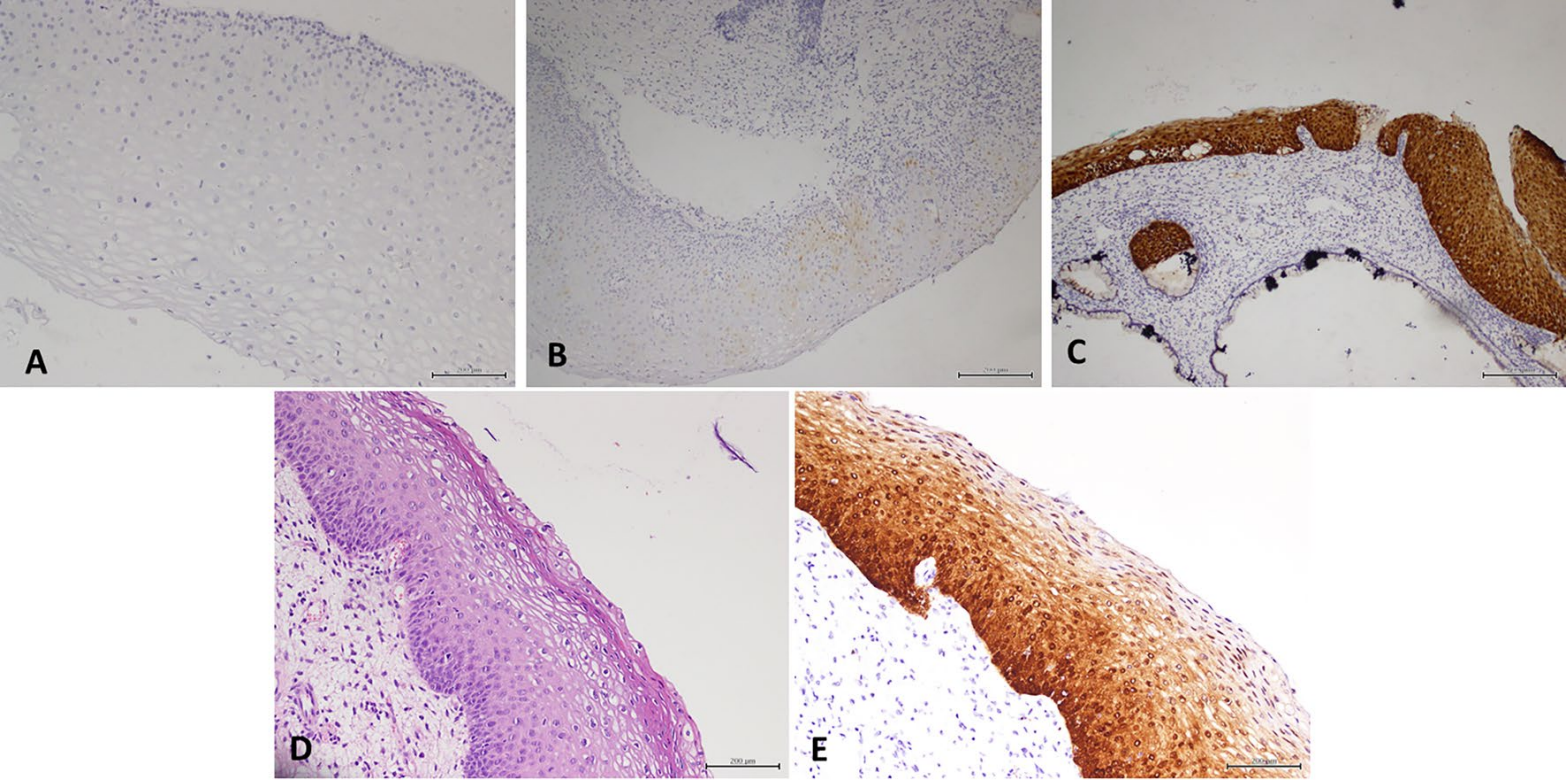
(**A**) P16 immunohistochemical staining showed negative (**B**) P16 immunohistochemical staining showed local weak positive(**C**) P16 immunohistochemical staining showed diffuse and strong positive (**D**) Histology shows the performance of LSIL (**E**) P16 immunohistochemical staining showed diffuse and strong positive over 1/3 layer of squamous epithelium.

According to the suggestion of the LAST project group ^1^, p16 positivity is defined as a continuous strong nuclear or nuclear plus cytoplasmic staining of the basal cell layer with extension upward involving at least one-third of the epithelial thickness. In our case, we found that p16 expression could be divided into three types: negative (Fig. 2A), weakly positive (Fig. 2B) and diffusely and strongly positive-extension upward involving at least one-third of the epithelial thickness (Fig. 2C). However, in our study, we found some cases with morphological features of LSIL that exhibited diffuse and strong p16 positivity (Fig. 2D–E).

**Figure 3:**
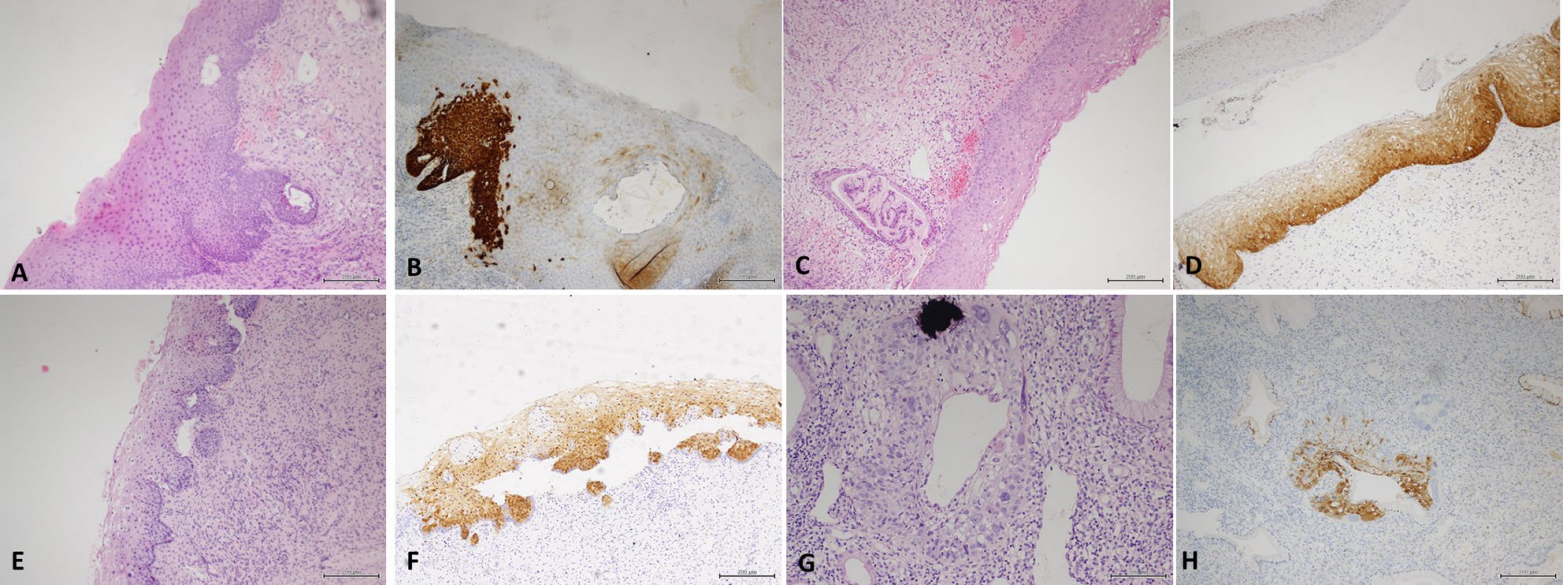
(**A**) Histologically interpreted as suspicious LSIL (**B**) P16 staining shows diffuse and strong positive staining in the lower part of the squamous epithelium (**C**) Morphology is difficult to determine whether it is LSIL or HSIL (**D**) P16 showed moderate positive in some areas and weakly positive in some areas (**E**) Histologically interpreted as LSIL (**F**) P16 shows uneven positivity of moderate intensity (**G**) The glandular epithelium showed obvious atypia (**H**) P16 shows uneven positivity of moderate intensity.

Furthermore, we encountered several cases of p16 staining that were difficult to interpret: ① in cases suspected to be LSIL by histology (Fig. 3A), p16 was mainly expressed in the lower one-third of the squamous epithelium and was diffusely and strongly positive (Fig. 3B). ② Morphology was difficult to determine regardless of whether the case was LSIL or HSIL (Fig. 3C); p16 was moderately positive in some areas and weakly positive in others (Fig. 3D). ③In cases histologically interpreted as LSIL (Fig. 3E), p16 demonstrated uneven positivity of moderate intensity (Fig. 3F). ④The glandular epithelium exhibited obvious atypia (Fig. 3G), and p16 showed uneven positivity of moderate intensity (Fig. 3H).

Among 1024 specimens, 349 were negative for p16 expression, 191 were weakly positive and 484 were diffusely and strongly positive.

Among 217 specimens whose histological diagnosis was normal, 158 were negative for p16 expression, 59 were weakly positive and 0 were diffusely positive. Among 68 specimens with a histological diagnosis of uncertain LSIL, 57 were negative for p16 expression, 9 were weakly positive and 2 were diffusely positive. Among 207 specimens with a histological diagnosis of LSIL, 118 were negative for p16 expression, 79 were focally positive and 10 were diffusely positive. Among 128 specimens with a histological diagnosis of uncertain HSIL, 12 were negative for p16 expression, 44 were weakly positive and 72 were diffusely and strongly positive. Among 404 specimens with a histological diagnosis of HSIL, 4 were negative for p16 expression, 0 were weakly positive, and 400 were diffusely and strongly positive (Table 1).

### The expression of Ki67 in various histological diagnosis situations

**Figure 4:**
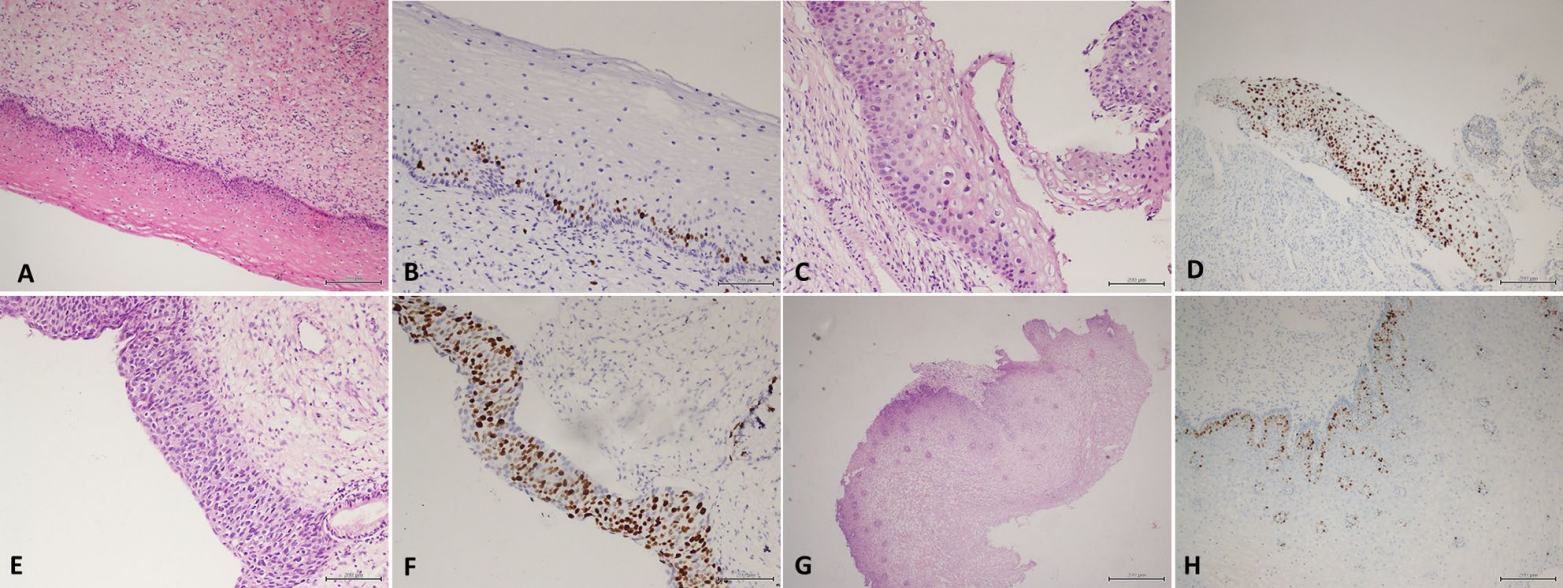
(**A**) Normal cervical squamous epithelium (**B**) Ki67 was mainly expressed in basal layer and parabasal layer in normal cervical squamous epithelium (**C**) Histological picture of LSIL (**D**) In LSIL ,Ki67 was not only expressed in basal layer and parabasal layer, but also expressed in 2 / 3 of squamous epithelium. (**E**) Histological picture of HSIL (**F**) In HSIL ,Ki67 was not only expressed in basal layer and parabasal layer, but also expressed in 2 / 3 of squamous epithelium. (**G**) Papillary hyperplasia of cervical squamous epithelium (H)In Papillary hyperplasia of cervical squamous epithelium, Ki67 was not only expressed in basal layer and parabasal layer, but also expressed in the axis of the vessel.

In our study, we found that Ki67 was primarily expressed in the basal and parabasal layers in the normal cervical squamous epithelium (Fig. 4A–B). However, in LSIL (Fig. 4C–D) and HSIL (Fig. 4E–F), Ki67 was expressed in the basal and parabasal layers and also in two-thirds of the squamous epithelium. When the squamous epithelium has papillary hyperplasia, Ki67 is also expressed around the vascular axis (Fig. 4G–H).

Among 217 specimens whose histological diagnosis was normal, Ki67 immunohistochemical staining was observed in the basal and parabasal layers in 209 cases and in the upper two-thirds of the squamous epithelium in 8 cases. Among 68 specimens whose histological diagnosis was uncertain LSIL, Ki67 immunohistochemical staining was observed in the basal and parabasal layers in 47 cases and in the upper two-thirds of the squamous epithelium in 21 cases. Among 207 specimens whose histological diagnosis was LSIL, Ki67 immunohistochemical staining was observed in the basal and parabasal layers in 15 cases and in the upper two-thirds of the squamous epithelium in 192 cases. Among 128 specimens whose histological diagnosis was uncertain HSIL, Ki67 immunohistochemical staining was observed in the basal and parabasal layers in 8 cases and in the upper two-thirds of the squamous epithelium in 120 cases. Among the 404 specimens whose histological diagnosis was HSIL, Ki67 immunohistochemical staining was observed in the basal and parabasal layers in 0 cases and in the upper two-thirds of the squamous epithelium in 404 cases (Table 1).

### Differences in p16 and Ki67 in different histological diagnoses and the diagnostic value of Ki67 for LSIL

Histological diagnosis is used as a standard reference. Therefore, we excluded those cases with uncertain histological diagnosis, including 68 cases of indeterminate LSIL and 128 cases of indeterminate HSIL. We used Fisher’s exact test to compare the differences in p16 expression between normal cervix and LSIL. We used the chi-squared test to compare the differences in p16 expression between LSIL and HSIL. We also used the chi-squared test to compare the differences in Ki67 expression between normal cervix and LSIL. Furthermore, we used Fisher’s exact test to compare the differences in Ki67 expression between HSIL and LSIL. The results indicated that p16 expression was significantly different among normal, LSIL and HSIL specimens. Ki67 expression was also significantly different among normal, LSIL and HSIL specimens (Tables 3 and 4).

**Table 3:** Differences in p16 and Ki67 immunohistochemistry between LSIL and normal cervix.

	Normal	LSIL	*P*-value
p16 (positive)	0	10	
p16 (negative)	217	197	0.001
Ki67 (Expression in the upper 2/3 layer)	8	192	
Ki67(Expressed in the basal and parabasal layers)	209	15	< 0.001

**Table 4:** Differences in p16 and Ki67 immunohistochemistry between LSIL and HSIL.

	LSIL	HSIL	*P*-value
p16 (positive)	10	380	
p16 (negative)	197	24	< 0.001
Ki67 (Expression in the upper 2/3 layer)	192	404	
Ki67(Expressed in the basal and parabasal layers)	15	0	< 0.001

## Discussion

The results of a study by Sagasta et al. indicated that p16 was diffusely positive in 230 LSIL/CIN1 lesions (45%), demonstrated focal positivity in 123 (24%) and was negative in 154 biopsies (30%); moreover, it has very low or no value as a marker of progression of LSIL/CIN1 in clinical practice^6^. Our study also confirmed that in a small number of LSIL cases, p16 immunohistochemical staining will be diffusely and strongly positive (10/207). Since the morphology supports LSIL, this may not lead to overdiagnosis of squamous intraepithelial lesions. However, in our study, some cases exhibited morphology between that of LSIL and HSIL. Most pathologists would likely diagnose these cases as HSIL (72/128). An article published by Maniar et al. suggests that 1/3 of CIN2 cases are diagnosed as CIN1 by some pathologists and that CIN1 cases are often not recommended for p16 immunohistochemical staining. When reviewing these cases and performing p16 immunohistochemical staining, most pathologists tend to make the diagnosis of HSIL only if p16 immunohistochemical staining is diffusely positive^7^. A retrospective study of a large number of cases of cervical squamous intraepithelial lesions showed that approximately 15% of patients with p16-positive LSIL immunohistochemistry would progress to HSIL, whereas 9.4% of patients with p16-negative LSIL immunohistochemistry would progress to HSIL. The authors believe that this difference is not enough to warrant differential management. In another large-scale case study, cervical squamous intraepithelial lesions were diagnosed by an expert team, and the patients were followed-up for a long period. The follow-up showed that regardless of the results of p16 immunohistochemical staining, LSIL diagnosed using morphology alone was more likely to subside. It is suggested that H&E morphology is still the most reliable method for the diagnosis of cervical squamous intraepithelial lesions^8^. Some studies have demonstrated that most (73.9%–100%) moderate (CIN2) lesions are p16-positive. However, because some CIN2 cases are negative for p16 via immunohistochemistry, the use of p16 staining to assist in the diagnosis of HSIL may lead to downgrading in nearly one quarter of cases^7^. In conclusion, there may be some problems with p16 in overdiagnosis or low diagnosis when used to assist in the diagnosis of squamous intraepithelial lesions.

Contrarily, p16 interpretation may be problematic. p16 positivity, as defined by the LAST team, is diffusely and strongly positive staining in more than one-third of the squamous epithelium. Liu et al. found that if p16 immunohistochemical staining did not completely show diffuse strong positive staining in the cytoplasm and nucleus, the diagnosis would be very difficult. These special cases include diffuse strong positive staining in the basal layer (8%), strong positive staining in a focal area (7%) and weak positive/strong positive staining in some areas (8%). These cases do not meet the diagnostic recommendations of p16 positivity proposed by the LAST project. It is worth considering whether these cases should be diagnosed as HSIL^9^. Such cases were also encountered in our study (Fig. 3d, 3f). In addition, cervical squamous intraepithelial lesions often appear in the transformation zone, and the glands may also exhibit morphological changes. In this case, the evaluation criteria of p16 positivity (strong positivity in more than one-third of the thickness) are not applicable to the diagnosis of HSIL (Fig. 3G–H).

Histologic assessment of cervical dysplasia is complicated by inter-observer variability that equals that of cytologic interpretation^10^. The most common problem is the distinction between LSIL and normal cervix. While not recommended by current management guidelines, women diagnosed with CIN1 are sometimes aggressively treated^5^. At present, no effective immunological marker of morphology is recognised to help in diagnosing LSIL. Ki67 expression in the middle and superficial one-third of the epithelium correlates well with the histopathological diagnosis of squamous intraepithelial lesions^11^. The high positivity rate of Ki67 immunohistochemistry suggests a high proliferation index and a high degree of malignancy. Ki67 immunohistochemical staining is of high value in distinguishing cervical squamous intraepithelial lesions from benign lesions (atrophic cervical squamous epithelium) because Ki67 exhibits higher expression in the former, but Ki67 staining cannot distinguish between dysplasia and immature squamous metaplasia^12,13^. Ki67 is expressed in the lower 1/3 layer of metaplastic epithelium, which suggests that these cells have strong proliferative activity. Some studies have also confirmed that Ki67 is related to the invasive ability of tumour lesions^5^. Other studies suggest that the Ki67 proliferation index is significantly increased in atypical squamous metaplasia, which may be due to the high proliferation rate of these cells caused by an inflammatory reaction^14^. Our study found that Ki67 is often expressed in the upper two-thirds of squamous intraepithelial lesions, which is helpful in distinguishing LSIL from cervix without HPV infection. The ROC curve suggests that Ki67 has a high diagnostic value for LSIL.

In clinical practice, p16 immunohistochemical staining is used to distinguish among morphologically indistinguishable cervical lesions (cin1 or cin2). However, both previous studies and our study suggest that there are some problems in the interpretation of p16 immunohistochemical staining in a few cases. For such difficult cases, new immune markers or other diagnostic methods need to be discovered in order to distinguish such lesions. In addition, our study suggests that Ki67 has a high diagnostic value in distinguishing LSIL from normal cervix. Therefore, we suggest that Ki67 can be used along with morphology for distinguishing LSIL from normal cervix. For patients whose biopsy results do not support LSIL, the frequency of reexamination can be reduced to reduce the financial and psychological burden on patients.

## Conclusions

In conclusion, Ki67 is often expressed in the upper two-thirds of squamous intraepithelial lesions. In cervical biopsy specimens, Ki67 can be used as an immunohistochemical marker to distinguish LSIL from cervix not infected by HPV. p16 can show diffusely and strongly positive expression in a small portion of LSILs. In addition, in some biopsy specimens, there may be some problems in explaining the positive expression of p16 in cervical squamous intraepithelial lesions, which may lead to variations between different observers.

## Data Availability

The datasets used and/or analyzed during the current study are available from the corresponding author upon reasonable request.
